# Competition Between Resonant Plasmonic Coupling and Electrostatic Interaction in Reduced Graphene Oxide Quantum Dots

**DOI:** 10.1038/srep36898

**Published:** 2016-11-22

**Authors:** Sanjay Karna, Meg Mahat, Tae-Youl Choi, Ryoko Shimada, Zhiming Wang, Arup Neogi

**Affiliations:** 1 Inst. of Fundamental and Frontier Sciences, Univ. of Electronic Science and Technology of China, Chengdu, P.R. China; 2 Department of Physics, University of North Texas, Denton 76203, USA; 3 Department of Mechanical and Energy Engineering, University of North Texas, Denton 76207, USA; 4 Department of Mathematical and Physical Sciences, Japan Women’s University, Tokyo, 112-8681 Japan; 5 Advanced Manufacturing and Materials Processing Institute, University of North Texas, Denton Texas, 76207, USA.

## Abstract

The light emission from reduced graphene oxide quantum dots (rGO-QDs) exhibit a significant enhancement in photoluminescence (PL) due to localized surface plasmon (LSP) interactions. Silver and gold nanoparticles (NPs) coupled to rGO nanoparticles exhibit the effect of resonant LSP coupling on the emission processes. Enhancement of the radiative recombination rate in the presence of Ag-NPs induced LSP tuned to the emission energy results in a four-fold increase in PL intensity. The localized field due to the resonantly coupled LSP modes induces n-π* transitions that are not observed in the absence of the resonant interaction of the plasmons with the excitons. An increase in the density of the Ag-NPs result in a detuning of the LSP energy from the emission energy of the nanoparticles. The detuning is due to the cumulative effect of the red-shift in the LSP energy and the electrostatic field induced blue shift in the PL energy of the rGO-QDs. The detuning quenches the PL emission from rGO-QDs at higher concentration of Ag NPs due to non-dissipative effects unlike plasmon induced Joule heating that occurs under resonance conditions. An increase in Au nanoparticles concentration results in an enhancement of PL emission due to electrostatic image charge effect.

Graphene oxide (GO) synthesized from the oxidation of graphite can contain sp^2^ carbon atomic cluster within the sp^3^ network^1^,^2^. The presence of the sp^2^ graphitic domains in GO can result in quantum confinement effects due to the formation of graphene oxide quantum dots. The optical properties and its dependency on the reduction level of GO indicate that the PL emission originates from the recombination within the small sp^2^ pure carbon clusters embedded within the sp^3^ network as well as the surface states of the carbon nanostructures^3^,^4^. Reduced graphene oxide has a significantly large exciton binding energy ~500 meV^5^, which can result in strong excitonic emission, optical nonlinear processes^6^ and coherent optical phenomena observable at room temperatures^7^,^8^.

The internal quantum efficiency of semiconducting GO emitter is rather low compared to other semiconductor light emitters irrespective of the synthesis techniques^9^,^10^. Efforts are recently directed towards increasing the emission from graphene oxide structures^6^. The ratio of the sp^2^ versus the sp^3^ cluster in a composite and the size of the sp^2^ clusters can influence the light emission properties^6^,^11^. We have used surface plasmon coupled to the green emission from graphene oxide to enhance the light emission efficiency^12^. However, the bandgap of the reduced GO (rGO) can be tuned from the UV to the infrared wavelength region^13^, which would require a broader range of metal plasmon frequency at the emission energy. The resonant coupling of long range surface plasmon to the emitted light is limited by the nature of the metal-dielectric interface. Tunable localized plasmons achievable by controlling the shape, size and the nature of the metal nanoparticles^14^ can be coupled to a range of emission from the rGO nanoparticles of various sizes or different reduction levels. Localized plasmons resulting in the enhancement of localized electric field due to the Ag nanoparticles can enhance the rate of reaction, electrical conductivity or result in photo-catalytic effects in graphene oxide^15^,^16^. Localized plasmons has utilized to enhance the light emission from CdSe based quantum dots^17^,^18^. Tuning the surface plasmon polariton resonance to the emission energy of the quantum dot can result in a significant enhancement^18^.

As these studies demonstrate that localized plasmons tuned to the emission energy of an emitter can enhance the light emission from semiconductor^18^,^19^ the present work presents a study of the plasmonic interaction that effects the optical properties of light emission from graphene oxide system^20^,^21^. Localized plasmons both resonant and off-resonant to the emission energy of the graphene oxide based nanoemitter is considered. The localized plasmon field is induced by Au or Ag nanoparticles and conjugated to reduced graphene oxide synthesized. It is observed that the photoluminescence (PL) enhancement depends strongly on the overlap of the localized plasmons with that of the emitted light. The strong coupling of excitons and plasmons under resonant plasmonic interaction is modified due to an increase in the density of nanoparticles around rGO nanoparticles. The increased concentration of Ag nanoparticles also results in an electrostatic effect that reduces the overlap between emission band of the emitter and the absorption band of the localized plasmonic modes and quenches the observed PL enhancement. The increase in concentration of Au nanoparticles that has localized plasmon energy coupled off-resonantly to the emission energy of rGO quantum dot emitters also results in PL enhancement due to image charge effect. Photoluminescence, and time resolved differential transmission spectroscopy techniques has been utilized to study the carrier recombination properties influenced by the localized plasmon field.

## Results

**Figure 1:**
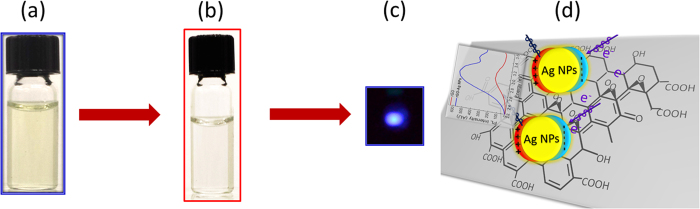
Schematic diagram shows PL emission from rGO samples under optical illumination (**a**) rGO, (**b**) rGO-Ag NPs, (**c**) fluorescence emission under the influence of a UV excitation at 300 nm and (**d**) Schematic of resonant Ag nanoparticles attached to reduced graphene oxide and observed PL enhancement.

Figure (1) illustrates the experimental observation of fluorescence emission from rGO with Ag NPs. A concentration dependence of the silver nanoparticles has been studied to obtain the optimum plasmonic density of state essential for the resonant enhancement of photoluminescence due to plasmons^21^. The concentration of Ag NPs in rGO was optimized as 0.003 mg Ag NPs per mL rGO solution. This concentration was optimized for maximum PL emission. The oxidation of graphite causes the formation of graphitic islands in GO which produces a disruption of the π-π* network and thus opens up a band gap in the electronic structure. The light emission is stable and the PL intensity does not change if the samples are not exposed to visible or UV light.

**Figure 2:**
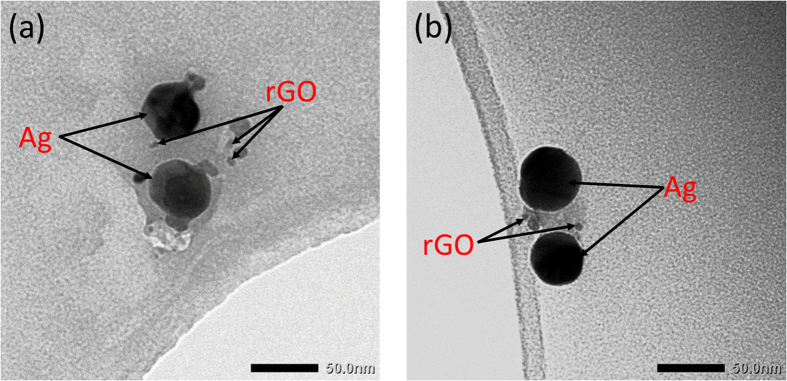
Figure shows a high resolution TEM image of Ag NPs on thin sheet of rGO along with embedded rGO NPs.

**Figure 3:**
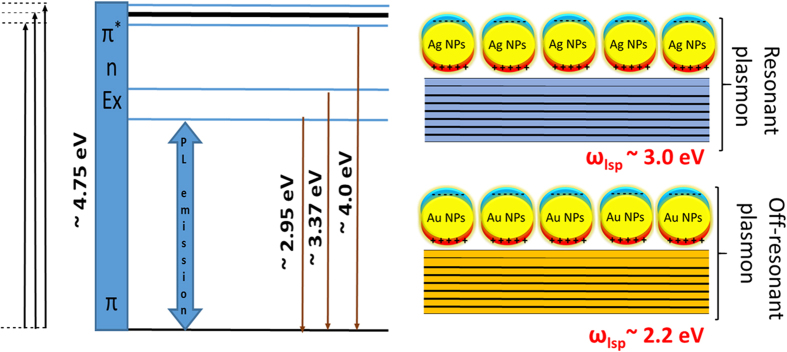
Schematic band diagram of rGO with the Au and Ag nanoparticles coupled for plasmonic interaction. The plasmon energy and the absorption and emission energy in the rGO is experimentally deduced from the spectroscopic measurements.

High resolution transmission microscope (HRTEM) images of rGO with Ag NPs shows silver nanoparticles on thin flakes of rGO with quantum dot like GO nanoparticles in the vicinity (Fig. 2). The Ag NPs are dispersive at the optimum concentration of 0.003 mg of Ag NPs/ml of rGO solution and avoid the possibility of agglomeration of Ag NPs^21^. These Ag nanoparticles are within the electrodynamic coupling distance of the rGO quantum dots or the monolayer flakes that emits light from both the quantum confined states and surface states in the carbon based material system^6^,^22^. The size of the metal nanoparticles was chosen specifically so that the LSP of Ag nanoparticles are close to the emission from the rGO. For studying the off-resonant plasmonic interaction of the Au nanoparticles was chosen such as the localized plasmon energy of Au nanoparticles are significantly lower than the absorption or emission edge of the rGO clusters. The resonant and off-resonant interaction is shown in the schematic band-line up in Fig. 3.

**Figure 4:**
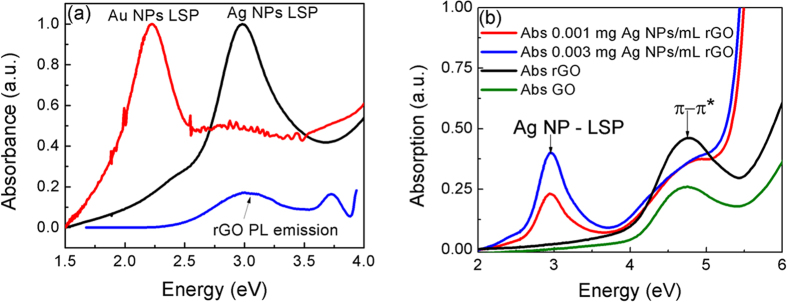
Figure shows the UV-vis absorbance of (**a**) rGO and Ag and Au NPs and emission from rGO (blue solid line) and (**b**) rGO with and without Ag NPs.

Absorption spectroscopy was performed to evaluate the localized plasmon energy of the metal nanoparticles ensemble as well as the transition levels in rGO nanoparticles using a UV-VIS absorbance spectrophotometer. In order to estimate the optimum excitation photon energy required for emission from a particular state, the excitation wavelength dependence of the photoluminescence emission was measured based on the absorption efficiency at various energy levels. The absorption spectra of the rGO suspension was measured in a 2 mm quartz cuvette within the spectral range of 200 to 900 nm. Figure (4a) shows the extinction spectra of metal nanoparticles compared to the underlying PL emission from the bare rGO sample without any nanoparticles. The silver nanoparticles (in black) has an extinction peak due to the localized plasmon at 2.98 eV (416 nm), whereas the gold nanoparticles (in red) has an emission at a relative longer wavelength at 2.2 eV. It has been observed that the Ag plasmon energy for these NPs extends from 1.84 eV to 3.65 eV. The Au Plasmon energy extends from 1.84 eV to 2.5 eV. A comparison of the localized surface plasmon peak energy of the metal NPs with the PL spectrum of rGO (shown in the next section) that the Ag plasmon peak overlaps resonantly with the emission from the rGO nanoclusters. Figure (4b) shows the change in absorption of the rGO attached with metal nanoparticles. The absorption at 4.74 eV in rGO is due to the π-π* transitions.

The absorption due to the n-π* transition lying in the 3.3 eV–3.4 eV are relatively much weaker^6^. Compared to rGO quantum dots, carbon quantum dots exhibit a well-defined n-π* absorption state. In the presence of the Au or Ag NPs, the absorption due to π-π* transitions is relatively reduced. There is no absorption of light in rGO within the range of Au plasmon energy. The absorption of the Ag-rGO NPs is dominated by the absorption or the scattering at the localized plasmon modes. Figure (4b) depicts that the absorption at 2.98 eV increases with an increase in its concentration in the rGO solution. The size distribution of sp^2^ cluster, number of layers per flake and surface functional groups on rGO are important factors influencing the absorbance^23^. The enhanced absorption due to the increased density of state due to LSP modes can provide more channels for an increase in the spontaneous emission rate which can result in an increased efficiency of the broadband fluorescence in the visible range^13^,^24^.

**Figure 5:**
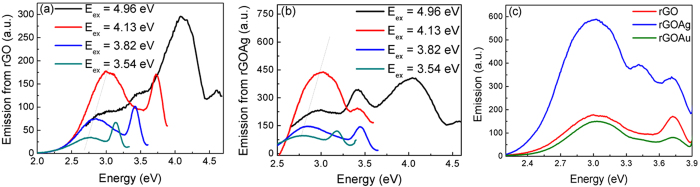
PL emission spectrum at various excitations energy from (**a**) rGO, (**b**) rGO with Ag NPs and (**c**) comparison of light emission from rGO (red solid line) and rGO with Ag (blue solid line) and Au NPs (green solid line) with the excitation at 4.13 eV.

The photoluminescence emission from rGO with and without Ag NPs at different excitations are shown in Fig. (5a,5b). The effect of excitation energy and nanoparticle concentration on the PL emission has been studied. An emission from the π-π* levels due to an excitation at 4.74 eV is observed at 4.11 eV. A sharper peak is accompanied at the higher energy side of the excitonic PL emission around 3.0 eV and is due to the Raman scattering of excitation light by the aqueous water solution. An excitation at 4.13 eV results in an excitonic emission at 3.0 eV bound to the surface of the rGO nanocluster. This emission overlaps with the localized plasmon energy as observed from the absorption spectrum of the Ag nanoparticles used in our experiments (Fig. 4a). It is observed that this emission at 3.0 eV redshifts with excitation energy. This red-shift indicates the re-graphitization process and implies the formation of new sp^2^ clusters^25^.

**Figure 6:**
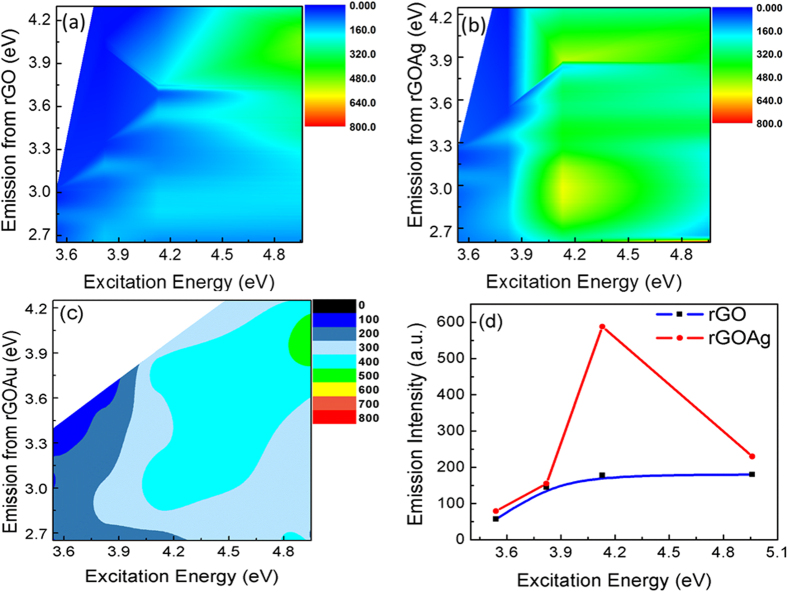
Contour plots exhibiting the excitation energy dependence of PL emission for (**a**) rGO NPs, (**b**) Ag NP- rGO nanoclusters, (**c**) Au NP- rGO nanoclusters and (**d**) the excitation energy dependence of PL emission for rGO-Ag NPs.

The effect of the localized plasmons on the emission from rGO is depicted in Fig. (5c). The PL emission at 3.0 eV is accompanied by a high energy peak at 3.35 eV due to the n-π* transition which is very weak and is not observed in bare rGO nanoclusters. The n-π* transition at 3.35 eV emission from rGO is induced by the resonant LSP modes due to Ag nanoparticles as the emission is not observed due to off-resonant LSP interaction in case of Au-rGO nanoclusters. It is also observed that the PL emission at 3.0 eV is enhanced by nearly 4 folds (Figs 5c and 6b,d) in the presence of the Ag nanoparticles when its LSP energy is resonant to the emission from rGO. Au nano-particles used in these experiments with LSP energy 2.2 eV was significantly lower than the emission energy of rGO (@ 3.0 eV) and thereby does not exhibit any enhancement in PL emission from rGO (Figs 5c and 6c). The enhancement of PL emission in rGO in the presence of Ag nanoparticles is due to an increase in the radiative recombination rate due to increased density of state at the resonant frequency.

The enhanced scattering of the PL emission due to the plasmon in the direction of detection could also lead to enhanced emission. The enhancement due to resonant plasmonic coupling has thereby been verified by energy excitation dependent emission measurements along with ultrafast time-resolved differential transmission measurement as the carrier relaxation rates are not influenced by directionality of the scattering process. The PLE spectral map shows that the maximum efficiency of emission in the rGO nanoclusters are due to the emission between the π-π* states, whereas in the presence of the Ag NPs-rGO system, the most efficient emission is observed from the states resonant to resonant LSP modes. There is also no shift in the emission energy from n-π* transition at 3.35 eV for various excitation energies (Figs 5b and 6b). It implies that the possible origin of emission at 3.35 eV is due to a strongly localized state coupled to localized plasmons. In Fig. (6b or 6c), the emission from the π-π* state in the ultraviolet range does not exhibit any enhancement as the localized plasmons energy due to Au or Ag NP is in the visible range and is significantly lower in energy than that of the emitted photons.

The excitation energy dependence of the PL emission enhancement is shown in Fig. (6d). At photon excitation energy below or above 3.82 eV, which corresponds to minima in the absorption spectrum in Fig. (4b), the PL emission intensity is significantly higher in the presence of localized plasmon modes induced by Ag NPs. At excitation corresponding to ~3.82 eV, the excited photon has minimal absorption due to the Ag nanoparticles but has sufficient absorption in rGO that most likely induces a n-π* transition resulting an enhanced PL efficiency at 3.0 eV due to the coupling of the emitted light in the presence of the localized plasmons.

## Discussions

**Figure 7:**
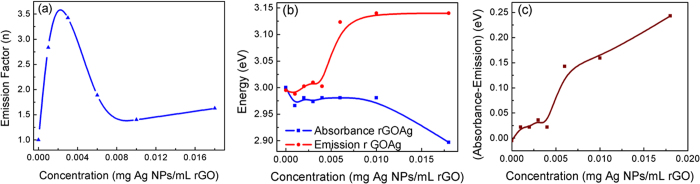
Ag nanoparticle concentration dependence in rGO (**a**) Enhancement of PL as a function of Ag nanoparticle concentration with excitation at 4.13 eV and PL emission at 3.0 eV, (**b**) Change in peak PL emission energy and integrated absorption intensity with concentration and (**c**) Stokes shift as a function of Ag nanoparticle concentration at the exctonic emission energy.

In order to study the limit or saturation of the PL enhancement, the dependence of the Ag-NPs concentration in the rGO complex is shown in Fig. (7a). The enhancement in the PL emission increases with the Ag nanoparticle concentration. However, for concentration exceeding 0.0032 mg Ag NPs/ml rGO, the PL enhancement decreases and at very high concentration, there is almost no enhancement in the integrated PL intensity (Fig. 7a). The PL enhancement factor is estimated from the ratio of PL intensity in the presence of Ag NPs to that of the emission intensity in the reference rGO samples of identical concentration.

**Figure 8:**
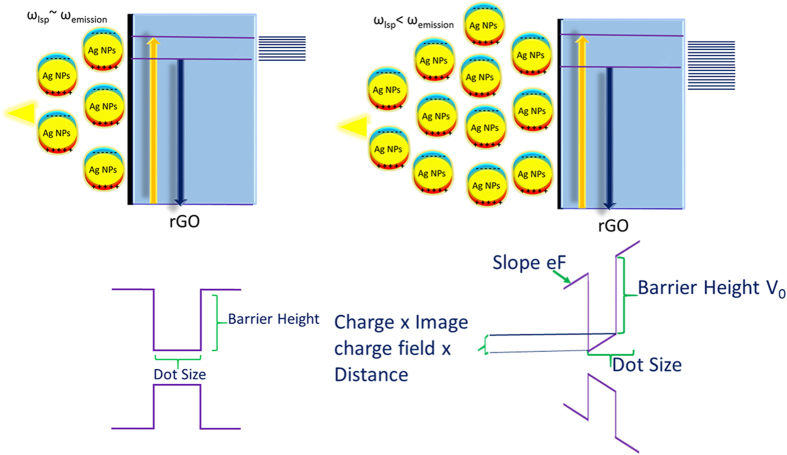
Schematic of the effect of increased concentration on the relative change in the bandedge of rGO due to plasmonic nanoparticles.

The Ag NPs concentration in most of the optical experiment is usually maintained at 0.003 mg Ag NPs per mL rGO. The enhancement in PL emission initially increases with the concentration of Ag NPs due to the increase in the photonic density of states around the emitter. At higher concentration the PL emission blue shifts and the absorption peak that was initially at 3.0 eV red shifts significantly due to aggregation of Ag NPs (Fig. 7b). This blue shift of the emission edge is due to the electrostatic Coulomb attraction induced by the formation of the image of the electrons and holes in the metal nanoparticles^26^. Figure (8) shows the electrostatic effects are prevalent when the radius of nanoparticles is less than 60 nanometers and the light is off-resonant compared to the localized plasmon energy. At resonance the plasmonic effects are dominant even though the electrostatic still exists. This Coulomb interaction results in a blue shift of the quantum confined bandgap due to image charge induced Stark effect. Conventional DC Stark effect usually leads to red-shift of the bandgap energy. However, electrostatic attraction of the electron and the holes with respect to their images formed in the metal nanoparticles results in a blue shift of the quantum confined bandgap^27^.

The red shift in absorption indicates is due to the decrease in the LSP energy due to the agglomeration of Ag nanoparticles in the presence of higher Ag NPs concentration (Fig. 7b)^21^,^25^. This results in a large Stokes shift which is more drastic at concentration above 0.0035 mg Ag NPs per mL rGO. The concurrent red-shift of the LSP absorption energy and the blue-shift the emission results in a reduced overlap of the electron and hole wave-function within the rGO nanostructures. This decreases the interband oscillator strength and results in decreased probability of the electron-hole recombination process.

Differential transmission spectroscopy provides an estimation of the photo-excited carrier life time. The plasmonic enhancement and the detuning of the plasmonic resonance from the bandedge emission of rGO is confirmed by time-resolved transient absorption spectroscopy. A comparison of the carrier lifetime and dephasing rates from energy states detuned from the bandedge due to off-resonant interaction in the presence of higher metallic nanoparticle concentration has been performed to demonstrate the effect of the image charge effect on the interband relaxation process. Transient absorption measurement has been carried out using an ultrafast Femtosecond Laser Source (Integra) by Quantronix seeded by a mode locked Ti: Sapphire laser (Mai-Tai) to generate a compressed laser output. The excitation laser energy was ~3.3 eV. The optical detection was carried out by a white light transient absorption spectrometer coupled with harmonic generator provided by ultrafast system. The source for the pump and probe pulses are derived from fundamental output of the Mai-Tai with 100 fs pulse width at a repetition rate of 1 kHz.

**Figure 9:**
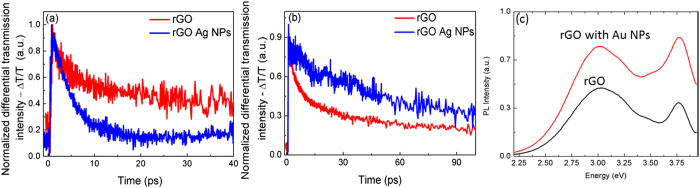
Differential absorption of rGO with and without Ag NPs (**a**) at resonance, (**b**) away from resonance (Ag NP concentration is 0.003 mg/mL of rGO) and (**c**) PL enhancement from rGO due to off-resonant coupling in the presence of Au nanoparticles (Au concentration of 0.005 mg Au NPs/mL rGO).

Time–resolved differential transmission measurement of rGO with and without Ag MNPs is shown in Fig. (9a). The traces show that the effective exciton life time of excited carriers in rGO decays faster in the presence of the resonant localized plasmon than rGO without Ag NPs on it. The faster decay time for rGO with Ag nanoparticles was attributed to the coupling of exciton energy with LSPs in the Ag nanoparticles. The effective carrier life time in rGO has two components, 2 ps due to carrier dephasing and 15 ps while the effective exciton life time of rGO with Ag NPS has only single component 4.5 ps. This single and faster decay time of Ag-rGO was attributed to the coupling of Ag plasmon with rGO emission energy. This faster decay time due to the coupling of Ag plasmon with rGO causing to enhance the emission intensity of rGO in PL measurement^28^.

Figure (9b) shows the differential transmission decay time for off-resonant interaction in a system with high Ag nanoparticle density. The quenching of emission is usually related to dissipative process such as carrier heating, Joule heating or phonon scattering which results in a decrease in the emitted light intensity. However, a longer life-time is observed in the case of Ag-rGO states beyond the strong coupling regime of resonant interaction. Under off-resonant conditions when the emission edge of the rGO emitter is blue shifted due to the image charge effect and the absorption edge is red-shifted due to plasmonics, the overlap integral between the electron and hole wave-function decreases which increases the carrier relaxation time compared to the situation when the image charge is weak. It also reduces the interband transition strength and results in a weaker band-band recombination. At resonance, the plasmonic effects dominate the image charge effect and results in an enhanced spontaneous emission rate, whereas away from the resonance, the image charge effect detunes the emission from the absorption edge resulting in a weak light emission.

Since, the electrostatic force between a charge and its image is always attractive, an isolated metallic nanostructure inclusion within a semiconductor attracts both electrons and holes equally, creating a boundary layer with a higher concentration of carriers. The Coulomb force due to the metal thereby catalyzes the modulation of carriers in the semiconductor. The charge carriers can be localized to a relatively narrow spatial region dependent on the range and dimension of inhomogeneous field around the metal nanoparticle. Within this ’enriched’ layer the probability of radiative electron-hole recombination may be orders of magnitude different than in the rest of the active zone of semiconductor emitter.

If the carriers in rGO may access the region in near vicinity of the metal, then within the rGO layer of thickness *d* the electrostatic energy of interaction of a carrier with its image is ~e^2^/ε*d*, where ε is the dielectric constant of the semiconductor. This energy must exceed kT, otherwise the electrostatic attraction is dominated by random thermal fluctuations. Assuming that ε ≈ 10 one obtains that at T = 300 K, the thickness of the layer d ≈ e^2^/εkT ~ 10 nm, which is comparable with the width of a single active layer of conventional multi-layered semiconductor light sources. This limit is valid when e-h plasma is in thermal equilibrium. In a light emitter the e-h plasma is not in equilibrium. In this case, the enriched layer is formed due to a dynamic balance between the recombining carriers, carriers created by external excitation, and carriers drifting resulting in dipole alignment towards the metallic inclusion due to attraction to their images in metal.

The off-resonant interaction under appropriate conditions can also result in an enhancement of PL emission which has been observed in case of GaAs quantum wells in the presence of Ga nanoparticles^26^. Although under lower concentration of Au nanoparticles, there is no enhancement of the emission from rGO, on increasing the concentration of Au nanoparticles coupled to the rGO quantum dots, the light emission intensity can be significantly enhanced.

In conclusion, the PL emission from rGO can be enhanced when the energy of the emitted photon in resonant to the localized plasmon energy of the metal nanoparticles. The PL enhancement also depends on the energy of the excitation and is optimum when the incident light energy is higher than the resonant localized plasmon energy modes but lower than the π-π* transition energy states. The PL enhancement also depends on the concentration of the Ag nanoparticles attached to the graphene oxide nanostructures. In the presence of high nanoparticle density, the electrostatic effect can result in a blue shift the emission from rGO and a red-shift in the absorption edge due to the plasmon mode owing to increased agglomeration. This results in detuning of the plasmon energy from the bandedge of the emission and reduces the enhancement significantly. The transient lifetime at resonant and off-resonant coupling process confirm the observed results. Short wavelength UV emission is also induced between the n-π* states due to resonant interaction of the resonant plasmon and is not observed in rGO system or in the absence of the plasmon coupled system. We have demonstrated strong spontaneous emission enhancement from graphene oxide nanoscale light emitters and the detrimental effect of competing electrostatic mechanism at high metallic concentration.

## Materials and Methods

**Figure 10:**
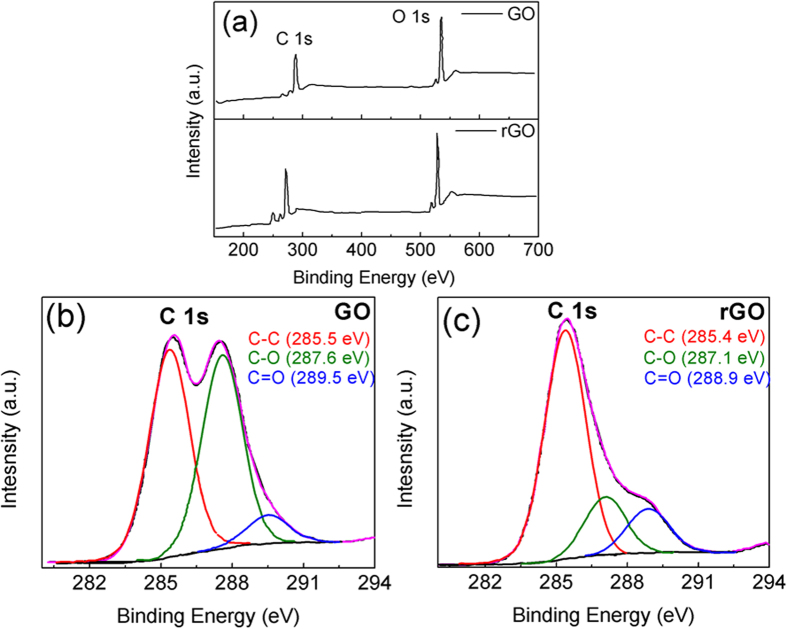
Showing XPS (**a**) spectra survey scan, deconvolution of C 1 s peak (**b**) in GO (**c**) in rGO.

Oxidized form of graphene i.e., graphene oxide (GO) was produced by oxidizing crystal graphite with a mixture of sulfuric acid (H_2_SO_4_), sodium nitrate (NaNO_3_), and potassium permanganate (KMnO_4_) by using the Hummers method^29^. According to this method, 1 gm of graphite powder was mixed with 23 mL of highly concentrated (~98%) of sulphuric acid at room temperature and then 0.5 gm of sodium nitrate added to the mixture. The mixture was cooled down to 0 °C and then treated with 3 gm of potassium permagnet. The solution was then maintained at 35–40 °C for half an hour and diluted with 47 mL of DI water and then 165 mL of DI water and further mixed with slow addition of hydrogen peroxide (H_2_O_2_) 30%. The mixture was filtered and repeated washing with hydrochloric acid (HCl) 1:10. The residual acids and salts were removed and then dried in freeze. The aqueous dispersion of few layer of GO can be obtained by using horn sonicator. The unexfoliated GO was removed by centrifugation at 4000 rpm. The homogeneous supernatant was obtained with complete exfoliation of almost all of GO sheets. After filtration dried under vacuum at room temperature. Structurally, Graphene oxide can be visualized as a graphene sheet with its basal plane decorated by oxygen-containing four groups such as hydroxyl, carboxyl, carbonyl and epoxy. Due to high affinity to water molecules by these groups, graphene oxide is hydrophilic and can be easily dissolved in water. Graphene oxide behaves as a semiconductor with its bandgap depending on the degree of reduction of graphene. It can be reduced by chemically, thermally and photo-catalytically and these processes yield varying degree of crystalline quality of carbon based nanostructure including GO based quantum dots^6^. The original solution of GO is composed of carbon 79% and oxygen 20% having flakes size of thickness of a few mono-atomic layer. Owing to the relatively high solubility of graphene oxide in de-ionized water, the GO solution is prepared in DI water. Graphene oxide complex of 0.005 mg/mL DI water is prepared and sonicated for 10 minutes. After sonication, the GO solution is reduced via photo-catalysis by exposing the solution to Xenon lamp for several hours. The GO solution was exposed by a Xenon lamp for 27 hrs for the reduction process which was determined to be optimum for the PL emission. The radiant power of the Xenon arc lamp was 50 watts. The rGO solution was treated with optimized concentration of silver or gold NPs in the rGO solution. It has been observed that further reduction in size of rGO QDs took place after treating with Ag NPs and that supports the possibility of photo-catalytic reduction in the presence of the Ag nanoparticles. During this process the removal of the various functional oxide groups occurred from high content of oxides in graphene oxide that leads the formation new sp^2^ cluster of various size. In the absence of any size separation techniques, the wide PL emission width does indicate polydispersity of the rGO nanoparticles. However, as the size distribution is rather large, the peak PL energy does not shift significantly in the presence of the Ag nanoparticles due to the chemical reduction. The energy shift occurs only when the metal concentration is rather high which leads to image charge induced blue shift in the emission spectra and agglomeration induced red-shift in the absorption spectra. The reduction level was confirmed by the strength of relative peaks in conventional micro-Raman spectrum of rGO materials. The reduction reaction of graphene oxide into graphene sheets was monitored by the color change from brown into black due to the absorption of visible light by large graphite or GO clusters. The concentration of the GO solution shown in Fig. (1a) is maintained very low concentration and so it doesn’t appear dark due to the dilution. The reduction time for UV light induced photo-catalysis was optimized to 27 hours to achieve the most stable rGO complex with higher emission efficiency. The reduction of the graphene oxide was further confirmed by X-ray photoelectron spectroscopy (XPS). Figure (10) shows the XPS spectra of GO and rGO and reveals the surface sensitive nature of the carbon atoms in rGO from the C1s spectra. A detailed analysis from the curve fitting of the XPS spectra indicates the peaks corresponding to the covalent bonds of carbon and oxygen atoms are more intense for GO than for rGO. The ratio of the intensity of the C-C and C = C bonds to that of the combinations of carbon and oxygen bonds is lower in GO clusters compared to that in rGO. The elementary composition of graphene oxide determined by XPS consisted of carbon, oxygen and hydrogen. As it is seen clearly from XPS analysis shown in Fig. (10a), the peak intensity of O 1 s reduces and C 1 s increases on reduction of graphene oxide. The peak area calculation of C and O elements from XPS spectral scan shown in Fig. (10a) reveals that the ratio of C and O atomic elements increased from 0.11 in GO to 1.1 in rGO. All the spectra were calibrated to the position of the C-1s peak of 285.5 ± 0.2 eV and the relative change in the reduction process was analyzed with respect to this peak energy. The XPS spectrum in Fig. (10a) was fitted for a comprehensive analysis of by using curve fitting after appropriate background correction. The high-resolution C1s signal was de-convoluted into three individual peaks of carbon atoms with bonding between carbon, hydrogen and oxygen atoms. The C1s spectra of GO and rGO are shown in Fig. (10b,c) respectively. The properties of graphene oxide depend on the variation of composition depending on the reduction level of graphene oxide. The oxygen content C-O in rGO decrease considerably from 44.3 At.% to 17.8 At.%. The removal of oxygen results in comparative abundance of C = O bonds in rGO which results in an increases in its atomic weight percent from 6.9 to 13.0. A quantitative analysis is shown in Tables 1 and 2 by using XPS. It can be inferred that the carbon amount increase (C-C, C = C bonds) and oxygen (C-O groups) decrease considerably on reduction.

Satellite peaks around the 285 eV was observed at ~275 and 265 eV. The origin of these species are not very clear but is likely due to S 2s^30^ or Cl 2p^31^ which are the impurities induced during the synthesis of graphene oxide^32^ using Hummer’s process that involves HCl, concentrated H_2_SO_4_, NaNO_3_, KMnO_4_ and H_2_O_2_. These peaks indicate the presence of impurities despite several filtration steps performed for the removal of traces of acid and salt. These impurities are less than 4.0 At.% and does not appear to influence the optical properties discussed in this report. This can be also inferred from a comparison of the line-widths of the absorption spectrum of GO and rGO in Fig. (4b). Considering the absorption cross-section of the rGO quantum dots and the excitation volume of the input light, the impurities does not affect the relatively fast plasmon mediated recombination process.

**Figure 11:**
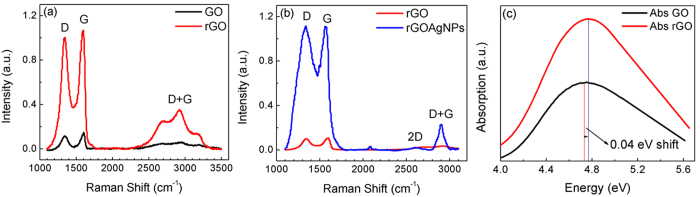
Optical measurement showing reduction of graphene oxide (**a**) Raman spectra of graphene oxide and reduced graphene oxide (**b**) Raman spectra of reduced graphene oxide (with and without Ag nanoparticles) (**c**) Absorption spectra of graphene oxide and reduced graphene oxide. These measurements confirm the size of sp^2^ cluster reduce and the formation of new sp^2^ cluster takes place on reduction of graphene oxide.

The Raman spectrum of GO and rGO has been shown in Fig. (11) for a comparative study of the change in the size of sp^2^ cluster due to reduction. A 532 nm laser excitation was utilized for the Raman spectroscopy measurements and the resulting laser frequency results in off-resonant Raman scattering. The laser also does not result in any excitation of localized plasmon modes within the Ag-rGO complex. Raman measurements shown in Fig. (11a) further demonstrate that the size of the rGO cluster is reduced and additional new sp^2^ clusters are produced. The GO solution was reduced over 27 hours by photocatalytic process for the maximizing the photoluminescence emission intensity. At different levels of reduction rGO QDs size was reduced and the PL intensity showed an increase. The decrease in the size of graphene oxide cluster due to reduction has also been analyzed using Raman analysis^33^,^34^. According to the Raman spectra of GO and rGO, the G band is due to in-plane vibrations of sp^2^ bonded carbon atoms whereas the D band is due to out-of-plane vibrations attributed to the presence of structural defects. On comparing the spectra of graphene oxide and reduced graphene oxide, rGO usually has a higher D band as shown in previous reports^33^ and is also observed in our present work Fig. (11a). This is due to the disruption of sp^2^ bonds of the carbon as GO has oxidative functional groups. Higher D band implies greater concentration of broken sp^2^ bonds. A higher intensity of D/G ratio implies an increase in distortion and this ratio is also inversely proportional to the sp^2^ cluster size^33^. In the present work, our calculation reveals that sp^2^ cluster size is 37.7 nm in GO which reduces to 24.34 nm in rGO. This Raman results conforms with the XPS results and demonstrates that there is a reduction of rGO. There is a significant enhancement of the Raman signal in the presence of the metal nanoparticles due to surface enhanced Raman scattering. The enhancement due to the Ag nanoparticles observed in the higher order Raman modes are significantly more than the enhancement of the fundamental modes. There are recent results reporting enhancement of higher order modes due to off-resonant plasmon enhanced structures^35^ showing similar enhancement.

The XPS and Raman results presented in Figs (10a–c) and (11a,b) are similar to results observed in reports related to the reduction of graphene oxide^30^,^31^,^36^,^37^,^38^. These results indicate the re-graphitization, removal of oxides components and size reduction after reduction of graphene oxide. The photocatalysis effect due to the presence of a localized plasmon at 3.0 eV results in further reduction of graphene oxide due to the presence of the localized field induced by the photoluminescence from the rGO emitters at 3.0 eV. A Raman analysis shows that the size of the rGO decreases from 24.34 nm to 19.82 nm in the presence of the Ag nanoparticles.

The surface charge was monitored using zeta potential measurement for the electrostatic attachment of metal nanoparticles. The zeta potential of the rGO nanoparticles synthesized nanoparticles was ~40 mV. Electrostatic coupling was used to attach the metal nanoparticle to the surface of the rGO nanoclusters. Silver nanoparticle (Ag NPs) of 40 nm in diameter and having a negative surface charge with 43.3 mV zeta potential off-resonant interaction can result in an enhancement which has been observed in case of GaAs quantum wells in the presence of Ga nanoparticles was mixed with the rGO nanoparticles in aqueous solution. Gold nanoparticles are also used as off-resonant plamonic material system that has a localized plasmon system which is significantly lower than the absorption or emission energy of the graphene oxide system. The zeta potential of the Au nanoparticles was ~40 mV.

In general, two main techniques for the synthesis of metal nanoparticle were used involving chemical and physical approaches. The most common for synthesis of Ag and Au NPs is a chemical method that was followed by reduction of organic and inorganic materials with suitable reducing agents. Various reducing agents were used for the synthesis of Ag nanoparticles. It included chemicals such as sodium citrate, ascorbate, sodium borohydride (NaBH_4_) for reduction of the silver ions (Ag^+^) in aqueous or non-aqueous solutions and chloroauric acid (H[AuCl_4_]) as a reducing agent for Au nanoparticles. These reducing agents reduce Ag^+^ and lead to the formation of metallic silver (Ag^0^) and similarly Au^3+^ions can be reduced to neutral (Au^0^). The reduction was followed by agglomeration into oligomeric clusters. These clusters eventually lead to the formation of metallic colloidal silver and gold nanoparticles. At these lower concentrations of rGO, the addition of Ag NPs in the solution makes the solution more transparent.

The optimized size of rGO QDs is 24.34 nm for the PL emission. At this optimized condition of rGO QDs size the PL emission enhanced has been observed by more than 3 times when treated with Ag NPs. The Ag NPs can also enhance the photo-catalysis rate in the presence of the localized plasmon field induced by UV-Visible irradiation from the xenon lamp^20^. The reduced absorption or scattering by particles comparable to the wavelength of the visible light makes the solution more transparent. In Fig. (11c), the blue shift in absorption peak indicates increased confinement and furthermore confirms reduction in the sp^2^ cluster size.

Optical Characterization: A SFPM RF-5301PC spectrophotometer was employed for the photoluminescence emission measurements. SFPM utilizes a UV enhanced xenon lamp source (150 W) with an excitation range of range 220–900 nm. The spectrophotometer is equipped with a 1300 lines/mm grating and has a detection range of 220–750 nm. A set of photomultipliers has been used for the detection of the photoluminescence from the sample. There are two detectors, one monitors the fluorescence from the sample and the other normalizes the incident light from the excitation source. The SFPM was used for excitation dependent synchronous emission spectrum measurement.

## Additional Information

**How to cite this article**: Karna, S. *et al*. Competition Between Resonant Plasmonic Coupling and Electrostatic Interaction in Reduced Graphene Oxide Quantum Dots. *Sci. Rep.* **6**, 36898; doi: 10.1038/srep36898 (2016).

**Publisher's note:** Springer Nature remains neutral with regard to jurisdictional claims in published maps and institutional affiliations.

## Figures and Tables

**Table 1:** XPS data of GO.

GO	Peak BE (eV)	At.%	Bond
C1s	285.5	48.8	C-C and C = C
C1s	287.6	44.3	C-O (epoxy, hydroxyl groups)
C1s	289.5	6.9	C = O (carbonyl groups)

**Table 2:** XPS data of rGO.

rGO	Peak BE (eV)	At.%	Bond
C1s	285.4	69.2	C-C and C = C
C1s	287.1	17.8	C-O (epoxy, hydroxyl groups)
C1s	288.9	13.0	C = O (carbonyl groups)
